# 非对称场流分离系统的构建及其在淀粉颗粒粒径表征中的应用

**DOI:** 10.3724/SP.J.1123.2021.05001

**Published:** 2021-11-08

**Authors:** Yuxi GUO, Tiange SONG, Yushan SUN, Qian YU, Haiyang DOU

**Affiliations:** 1.河北大学基础医学院, 河北 保定 071000; 1. College of Basic Medical Sciences, Hebei University, Baoding 071000, China; 2.河北大学化学与环境科学学院, 河北 保定 071002; 2. College of Chemistry and Environmental Science, Hebei University, Baoding 071002, China

**Keywords:** 非对称场流分离, 淀粉颗粒, 粒径表征, asymmetrical flow field-flow fractionation (AF4), starch granule, size characterization

## Abstract

淀粉颗粒粒径与分子尺寸分别在1~100 μm和20~250 nm之间,是影响淀粉功能特性的重要因素之一。非对称场流分离(AF4)是一种基于样品与外力场相互作用机制的分离技术,已应用于表征淀粉分子尺寸分布。商品化的AF4系统的粒径检测范围为1 nm~10 μm,对于淀粉颗粒粒径表征具有一定的局限性。该文研制了AF4分离系统;考察了其在微米尺度下对红薯、莲子和大米淀粉颗粒粒径表征的性能;采用微米尺寸的聚苯乙烯乳化球(PS)标准样品验证了构建的AF4系统的分离性能。实验结果显示,构建的AF4系统对PS混合样品(粒径2、6、12、20 μm)实现了基线分离,同商品化AF4相比提高了检测上线,具有分离表征淀粉颗粒的潜力。此外,该文研究了载液组成对淀粉颗粒分离表征的影响;通过光学显微镜验证了构建的AF4系统在微米尺度上对淀粉颗粒粒径分布的表征能力。最后,采用商品化的AF4系统串联多角度激光光散射检测器和示差折光检测器对3种淀粉分子进行了分离表征,考察了淀粉的溶解温度对其表征结果的影响。在摩尔质量10 ^6^~10^8^ g/mol范围内,红薯和莲子淀粉的回转半径和水合半径的比值(*R*_g_/*R*_h_)在0.9~1.1之间,大米淀粉的*R*_g_/*R*_h_在1.2~1.4之间。实验结果证明构建的AF4系统是一种快速、准确的淀粉颗粒粒径表征方法,与商品化的AF4系统结合可为研究淀粉尺寸分布与其功能性质之间的关系提供技术支持。

## Abstract (en)

Starch occurs naturally in the form of semicrystalline granules, and is composed of two types of carbohydrate molecules, amylose (AM) and amylopectin (AP). Starch granules and starch molecules have sizes in the range of 1-100 μm and 20-250 nm, respectively; these size ranges are among the key factors affecting the functional properties of starch. Asymmetrical flow field-flow fractionation (AF4) is a size-based separation technique. The major difference between AF4 and dynamic light scattering or microscopy techniques is that AF4 enables the separation of particles based on their size; consequently, the elution profile can be converted to the size distribution of the samples. In the last two decades, AF4 systems, when coupled online with multiangle light scattering (MALS) and differential refractive index (dRI) detectors (AF4-MALS-dRI), have demonstrated to be applicable for the size characterization of starch at the molecular level. Unlike size exclusion chromatography (SEC), AF4 systems use an open channel that does not require a stationary phase or packing materials. Thus, the shear scission of AP molecules during AF4 separation is minimized. The size detection range of a commercial AF4 system ranges from 1 nm to 10 μm, which is smaller than the size range of starch granules. In this study, a home-made AF4 system was developed, and its capability for the size characterization of starch granules extracted from sweet potato, lotus seed, and rice was investigated. The performance of the developed AF4 system was evaluated by running a mixture of polystyrene (PS) with diameter of 2, 6, 12, and 20 μm, respectively. Baseline separation of four PS samples was achieved, and the resolution for 6 μm PS and 12 μm PS was 1.40. The detection limit of the developed AF4 system was higher than that of commercial AF4 systems. Thus, the developed AF4 system is promising for the separation and characterization of starch granules. The effect of the composition of the carrier liquid on the AF4 separation of starch granules was also studied. Moreover, the accuracy of AF4 in terms of size characterization of the starch granules was evaluated by optical microscopy (OM). The results revealed that the type of dispersant and viscosity of the carrier liquid affect the accuracy of size characterization of the starch granules. The size distribution of rice starch granules obtained using a carrier liquid containing 0.01% (w/v) sodium dodecyl sulfate (used as a dispersant), 0.02% (w/v) NaN_3_ (used as a bactericide), and 0.001% (w/v) hydroxypropylmethylcellulose (used to adjust the viscosity of the carrier liquid) was in agreement with that obtained from OM. Furthermore, a commercial AF4 system coupled with MALS and dRI detectors was employed for the separation and characterization of starch molecules. A molecularly dispersed solution is necessary for the reliable molecular characterization of starch. The effect of the starch dissolution temperature on the AF4 characterization of starch was also investigated. The optimal dissolution temperature for lotus seed and rice starch granules was 75 ℃, while that for sweet potato starch granules was 78 ℃; this difference is mainly attributed to the different botanical origins of the granules. The results showed that the ratio of the radius of gyration (*R*_g_) to the hydrodynamic radius (*R*_h_) of rice starch and sweet potato starch is in the range of 0.9-1.1 over the molar mass range of 10 ^6^-10^8^ g/mol. For rice starch, the *R*_g_/*R*_h_ ratio is between 1.2 and 1.4. Rice starch has the highest apparent density among the three starches, indicating that rice starch molecules have a dense structure. The results demonstrated that the AF4 system developed in this study is rapid and accurate for the size characterization of starch granules. The developed AF4 system, when combined with commercial AF4 systems coupled online with MALS and dRI detectors, can provide technical support to study the relationship between the size from the nanoscale to the microscale and functional properties of starch.

淀粉是人类主要的能量来源之一,以其独有的物理化学性质和功能特性广泛应用在食品、医药等领域。在食品生产中,淀粉作为一种重要的食品配料,对于调整食品的质地有重要作用^[1,2]^。淀粉成膜性和冻融稳定性被应用于胶凝剂、增稠剂,稳定剂和涂层剂等工业^[3,4]^。淀粉颗粒大小是影响其功能特性的重要因素之一^[5]^。目前,表征淀粉颗粒大小常用的方法有动态光散射技术(dynamic light scattering, DLS)和显微镜技术^[6]^。DLS分析样品时间短、操作简单、成本低,但对于粒径分布较宽的样品,不同粒径颗粒的散射光之间会产生干涉,降低样品粒径表征结果的准确性^[7]^。光学显微镜(optical microscopy, OM)操作简单、分析成本低,可以同时提供淀粉颗粒形貌及粒径分布信息,但OM检测的样本数量有限,分析时间长。

20世纪60年代Giddings博士^[8]^首次提出场流分离(field-flow fractionation, FFF)概念,其中非对称场流分离(asymmetrical flow field-flow fractionation, AF4)是一种应用最广的FFF子技术,无固定相和填充材料,适用于剪切力敏感的样品的分离,检测范围广(1 nm~50 μm)^[9]^。该技术已应用于蛋白质及其复合物、纳米粒子、亚细胞单元和聚合物的分离表征^[10]^。AF4可与多角度激光光散射检测器(MALS)和示差折光检测器(dRI)联用(AF4-MALS-dRI),表征淀粉分子的粒径分布^[11]^。目前,商品化的AF4系统检测上限为10 μm,对于淀粉颗粒粒径表征具有一定局限性。本研究构建了AF4系统,结合商品化的AF4系统,与紫外可见光检测器(UV)、MALS和dRI联用,考察了其在纳米与微米尺度下对淀粉分离表征的性能。

## 1 实验部分

### 1.1 仪器、试剂与材料

SQP电子天平(北京赛多利斯科学仪器有限公司,中国); MS-H550-Pro磁力搅拌器(北京大龙兴创实业有限公司,中国); DHG-914385-Ⅲ电热鼓风干燥箱(上海新苗医疗器械制造有限公司,中国); Eclipse Ci型显微镜(尼康公司,日本); ZEN 3700型动态光散射粒度分析仪(马尔文公司,英国); UPR-Ⅱ-10T超纯水系统(西安优普仪器设备有限公司,中国); LC-20AT液相泵和SPD-20A紫外可见光检测器(岛津公司,日本); DAWN EOS MALS型多角度激光光散射检测器(怀雅特公司,美国); 1260 Infinity Ⅱ示差折光检测器(安捷伦公司,德国)。

大米、莲子、红薯购自当地超市;马脾铁蛋白、溴酚蓝(BPB)购自美国Sigma-Aldrich公司;聚苯乙烯乳化球(PS)、FL-70购自美国Thermo Fisher公司;NaOH、HCl、NaHSO_3_、SDS(sodium dodecyl sulfate)、NaNO_3_、Triton X-100(polyethylene glycol tert-octylphenyl ether)、NaN_3_、羟丙基甲基纤维素(HPMC)购自上海麦克林生化科技有限公司。

### 1.2 实验方法

1.2.1 淀粉颗粒及淀粉溶液的制备

淀粉颗粒的制备:将大米、红薯和莲子洗净晾干。根据Schoch等^[12]^描述的方法提取淀粉颗粒。具体方法如下:室温下,将40 g大米在200 mL NaHSO_3_(1 g/L)中浸泡12 h后使用厨房料理机在250 W的条件下粉碎2 min,依次通过80和120目筛,将滤液转入2 L烧杯中,静置5 h后弃上清液,然后用去离子水重悬沉淀物,静置3 h,重复3次,沉淀物在40 ℃的烘箱中干燥得到淀粉颗粒。

淀粉溶液的制备:室温下,将15 mg淀粉颗粒和1.5 mL 1 mol/L NaOH添加到20 mL玻璃瓶中,200 r/min搅拌6 min。然后将装有大米、莲子淀粉的玻璃瓶放置在75 ℃的水浴中,装有红薯淀粉的玻璃瓶放置在78 ℃的水浴中,400 r/min搅拌4 min,加入4.5 mL去离子水,继续搅拌2 h。最后加入1 mol/L HCl调节pH至7,得到淀粉溶液。

1.2.2 光学显微镜对淀粉颗粒的表征

将10 μL淀粉悬浮液(3.0 g/L)滴到显微镜载玻片上,在400倍的放大倍率下观察淀粉颗粒的形貌与大小(Nikon Digital Sight软件)。由于淀粉颗粒形状不规则,本实验所测长度为平面下颗粒的最长尺寸。每种淀粉每次统计500个颗粒,得到其平均直径,所有检测重复3次。

1.2.3 Zeta电位的表征

样品检测质量浓度为3.0 g/L,采用动态光散射粒度分析仪对不同载液条件下的淀粉颗粒悬浊液的Zeta电位进行分析,所有检测重复3次。

1.2.4 非对称场流分离系统的构建及对淀粉颗粒粒径的表征

AF4系统构建及对淀粉颗粒的分析:采用10 kDa再生纤维素(RC)膜和350 μm垫片组装的AF4通道构建了AF4系统(见图1)。通道具有梯形的几何形状,长度为17.4 cm,入口和出口的宽度分别为2.2 cm和0.15 cm。通过BPB确定聚集时间,通过铁蛋白确定通道实际厚度为290 μm。载液为0.01%(w/v)表面活性剂和0.02%(w/v)NaN_3_的去离子水。将20 μL淀粉颗粒悬浮液以0.2 mL/min的速度注入通道。检测器流速和交叉流流速分别为1.0和0.3 mL/min。通过SPD-20A紫外可见光检测器在254 nm波长下对淀粉颗粒进行检测。

**图1:**
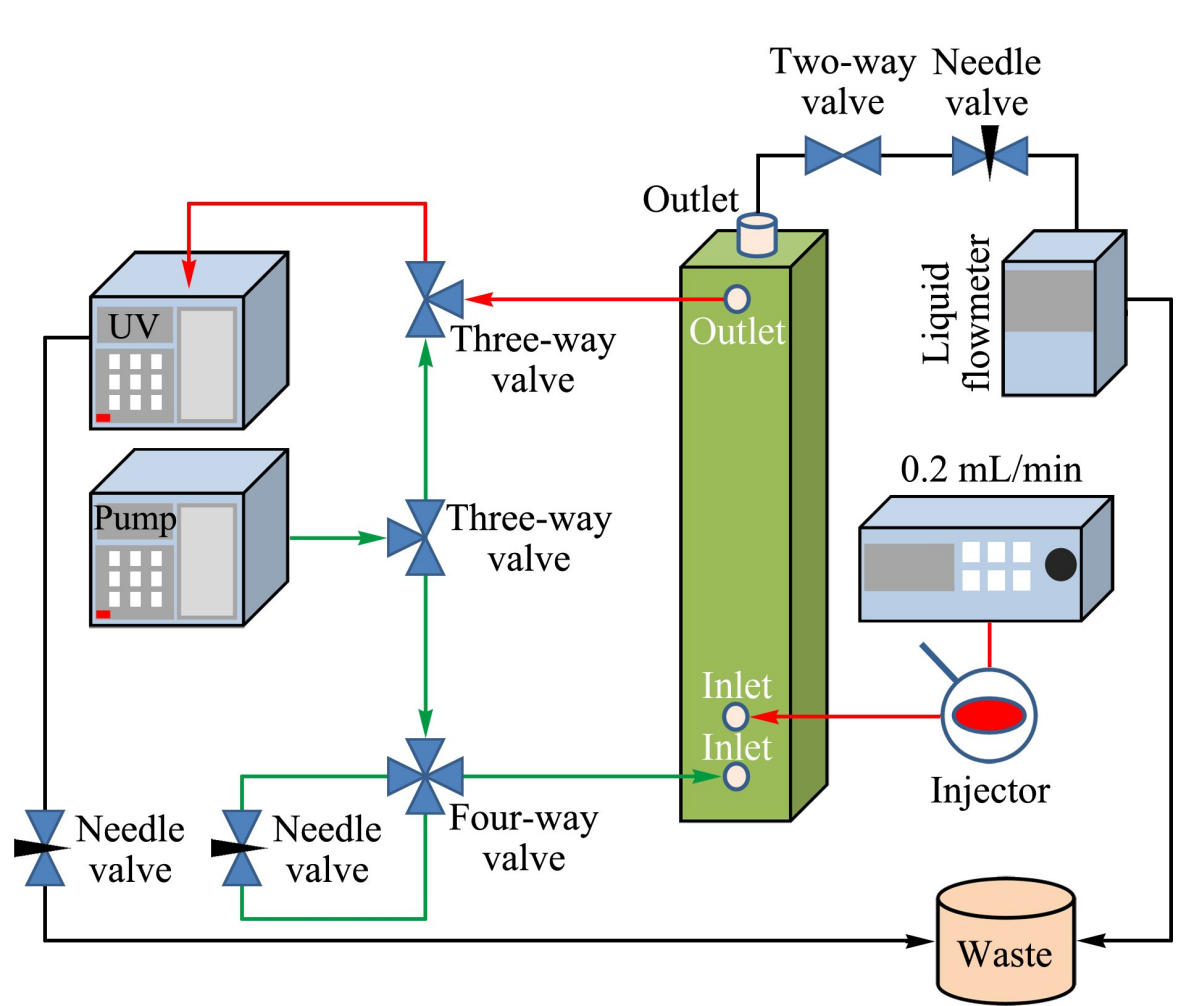
构建的AF4系统示意图

AF4在空间位阻模式下的保留时间(*t*_r_)和样品的水合直径(*d*)有如下关系^[13]^:


(1) log *t*_r_=-*S*_d_ log *d*+log *t*_r1_

*t*_r1_为具有单位直径的粒子的*t*_r_的外推值;*S*_d_为样品颗粒的分离度。

斜率(*S*_d_)和y轴截距(*t*_r1_)由校准曲线确定后,根据公式(1)可将AF4-UV洗脱图谱(*c*(*t*_r_))转换为粒径质均分布(*m*(*d*))^[14]^:


(2) $m(d)=c\left(t_{\mathrm{r}}\right) V S_{\mathrm{d}} t_{\mathrm{r} 1}\left(\frac{t_{\mathrm{r}}}{t_{\mathrm{r} 1}}\right)^{\frac{S_{\mathrm{d}^{+1}}}{S_{\mathrm{d}}}}$

*V*为体积流速。粒径数均分布(*n*(*d*))^[15]^:


(3) $n(d)=\frac{m(d)}{d^{3}}$

淀粉溶液的分析:使用商品化的AF4系统,载液为5 mmol NaNO_3_的去离子水溶液,样品浓度为0.30 g/L,进样流速为0.20 mL/min,进样体积为50 μL,交叉流流速从1.20 mL/min指数下降到0.05 mL/min,半衰期(*t*_1/2_)为3.0 min。通过BPB确定聚集时间,通过铁蛋白测得通道实际厚度为287 μm。

## 2 结果与讨论

### 2.1 构建的AF4系统的分离性能

采用微米尺寸的PS标准样品对构建的AF4系统分离性能进行了评估。检测器流速为1 mL/min,交叉流速为0.3 mL/min,选择载液为0.02%(w/v)FL-70和0.02%(w/v)NaN_3_去离子水溶液,按体积比1:1:1:1混合2、6、12和20 μm粒径的PS标准样品,进行AF4分离性能检验(见图2a)。实验结果显示,构建的AF4系统对混合的4种微米级PS标准样品实现了基线分离,2和6 μm粒径的PS分辨率为3.65, 6和12 μm粒径的PS分辨率为1.40, 4种PS标准样品的校准曲线*R*^2^为0.999(见图2b)。实验结果证明了构建的AF4系统对微米级样品具有良好的分辨率,同商品化AF4相比,其检测上限更高,具有分离表征淀粉颗粒的潜力。

**图2:**
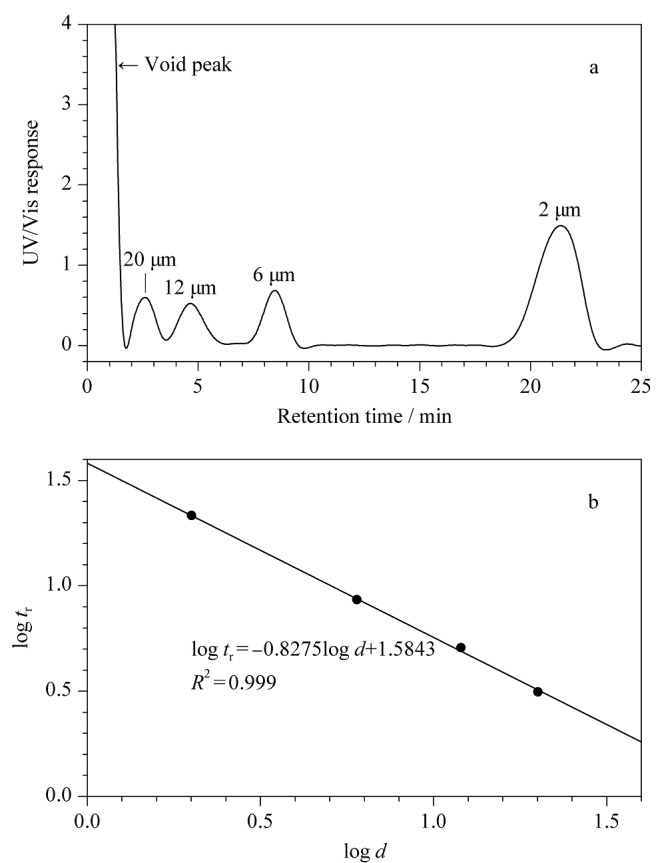
聚苯乙烯(PS)标准样品的(a)AF4洗脱谱图和 (b)校准曲线

### 2.2 构建的AF4系统表征淀粉颗粒粒径的分布

2.2.1 载液组成对构建的AF4分离表征淀粉颗粒粒径的影响

载液组成是影响样品与AF4超过滤膜交联的主要因素之一,根据不同样品表面性质的差异,需采用不同的表面活性剂分散样品颗粒。本文考察了3种表面活性剂对AF4分离表征淀粉颗粒的影响。载液为0.01%(w/v)表面活性剂和0.02%(w/v)NaN_3_(抑菌剂)的去离子水溶液。当交叉流流速为0 mL/min,直接进样品得到的峰面积为*A*_0_;当交叉流流速为0.3 mL/min,样品峰面积为*A*_1_,淀粉颗粒的回收率为*A*_1_/*A*_0_。不同载液条件下,AF4分析3种淀粉颗粒的回收率列于表1。实验结果表明,3种淀粉颗粒在SDS载液中的回收率最高;其中大米淀粉颗粒的回收率为98.89%。根据Plocková等^[15]^报道,在AF4分析过程中,样品在不同成分载液中的回收率差异可用静电力解释。

**表1:** 不同载液条件下AF4分析淀粉颗粒样品的回收率(*n*=3)

Carrier liquid	Sample recoveries/%		
	Sweet potato	Lotus seed	Rice
Water	41.99±0.02	14.98±0.02	82.76±0.01
Polyethylene glycol tert-octyl-	75.95±0.17	57.30±0.05	83.13±0.01
phenyl ether (Triton X-100)			
Sodium dodecyl sulfate (SDS)	94.03±0.02	80.47±0.03	98.89±0.04
FL-70	88.77±0.03	40.98±0.31	77.13±0.11

中性条件下,AF4超过滤膜表面主要带负电荷,增加样品表面负电荷可提高样品与AF4超过滤膜之间的静电排斥力,减小样品与超过滤膜的交联,进而提高样品的回收率^[16]^。Zeta电位表征结果显示(见表2), 3种样品在所有载液条件下带负电,相同来源的淀粉颗粒样品在SDS载液中的Zeta电位绝对值最大;在SDS载液中,莲子淀粉表面电位绝对值(45.33±1.36)大于大米淀粉表面电位绝对值(25.9±0.53),但是莲子淀粉颗粒的回收率小于大米淀粉颗粒,说明Zeta电位不是影响样品与超过滤膜表面交联的唯一因素。载液黏度与样品的结构特性共同影响样品与超过滤膜表面的交联^[17]^。

**表2:** 不同载液条件下淀粉颗粒的Zeta电位(*n*=3)

Carrier liquid	Zeta potentials		
	Sweet potato	Lotus seed	Rice
Water	-24.10±0.72	-19.97±0.64	-17.93±1.57
Triton X-100	-26.80±0.60	-24.63±0.35	-16.93±0.81
SDS	-35.97±1.10	-45.33±1.36	-25.90±0.53
FL-70	-30.43±0.74	-35.20±0.36	-20.07±0.55

2.2.2 构建的AF4系统表征淀粉颗粒粒径的准确性

采用OM检验构建的AF4系统表征淀粉颗粒粒径的准确性。OM测得的淀粉颗粒粒径分布如图3柱状图所示。实验结果显示,红薯、莲子、大米淀粉颗粒的尺寸范围分别为3~32 μm、2.1~23.5 μm、1.5~12 μm;平均粒径分别为12.16±0.11 μm、16.04±0.79 μm、5.25±0.30 μm。根据公式(1)~(3),结合图2的PS标准曲线,将不同载液条件下得到的淀粉颗粒的AF4洗脱图谱转换成粒径数均分布(见图3),平均粒径列于表3。实验结果显示,AF4测量淀粉颗粒平均粒径整体小于OM测量值。这主要是因为AF4测量的是样品水合直径,对于不规则样品,OM采用颗粒的最长尺寸为直径。此外,实验结果显示,在纯水条件下,AF4测量的莲子淀粉颗粒平均粒径(11.05±0.54 μm)小于OM测量值,这可能是由于在此条件下莲子淀粉的回收率为14.98%(见表1),只有部分莲子淀粉颗粒被检测到,导致OM测量的粒径分布代表性差。在SDS载液条件下,AF4测得的大米淀粉颗粒粒径大于OM测量值。这可能是由于淀粉颗粒在AF4空间模式中的洗脱受重力、升力、外加力和静电排斥力影响,升力与静电排斥力之和大于外加力与重力之和,使淀粉颗粒更接近池道中心,洗脱速度更快,保留时间更短,进而和图2得到的PS标准曲线有偏差,所以大米淀粉在SDS载液条件下洗脱时,AF4测量平均粒径值比OM测量值大。Woo等^[18]^报道:载体黏度影响PS的保留率,当载液黏度接近1时,通道底部与颗粒之间具有相互排斥作用并随载液黏度的增加保留率先下降后上升。已知载液中只含有0.01%SDS载液时,黏度接近于1。为增加载液黏度,减小样品所受的静电排斥力,在0.01%SDS+0.02%NaN_3_载液中加入0.001%(w/v)HPMC,使样品更接近AF4通道底部,相应结果见图4。在此条件下,AF4测量大米淀粉颗粒的数均分布与OM测得结果相似。

**图3:**
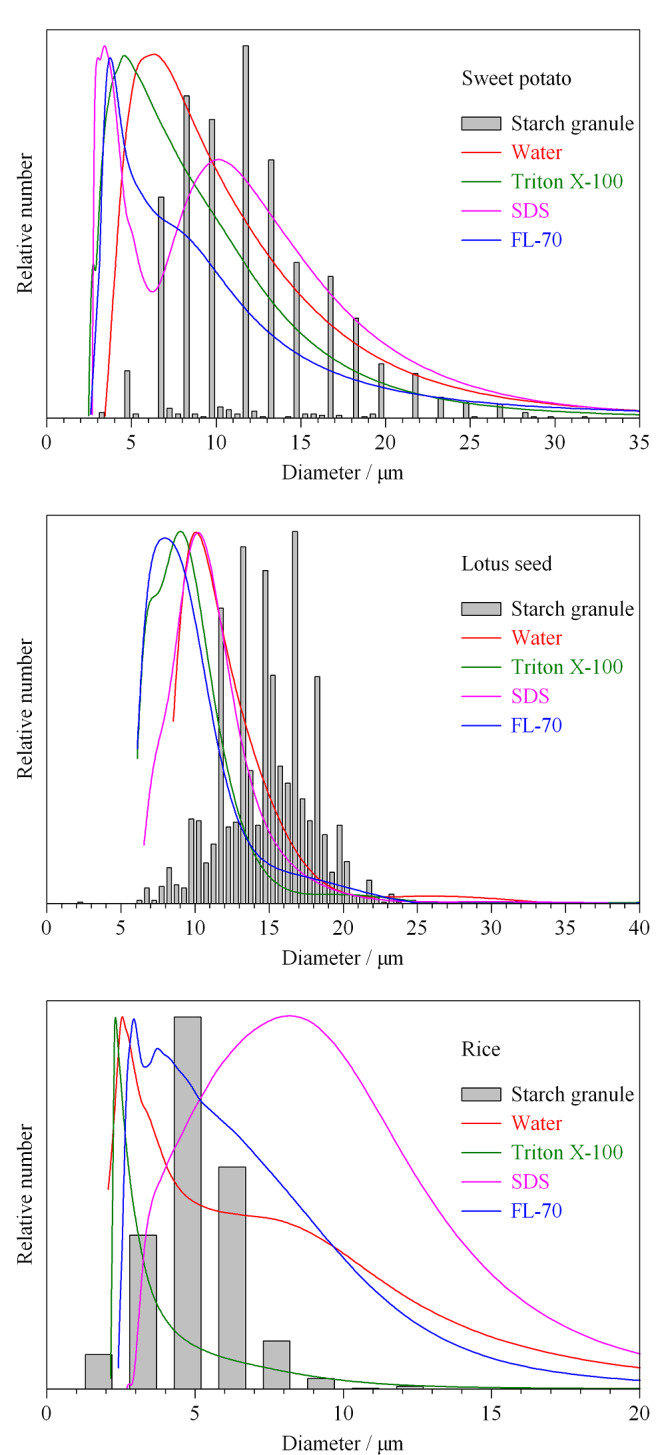
不同载液条件下淀粉颗粒的粒径数均分布

**表3:** 不同载液条件下AF4表征淀粉颗粒平均粒径结果(*n*=3)

Carrier liquid	Mean diameters/μm		
	Sweet potato	Lotus seed	Rice
Water	7.02±0.43	11.05±0.54	4.30±0.49
Triton X-100	5.23±0.42	8.68±0.32	3.50±0.06
SDS	10.13±0.19	9.63±0.18	6.74±0.33
FL-70	4.36±0.07	8.66±0.02	4.49±0.25

**图4:**
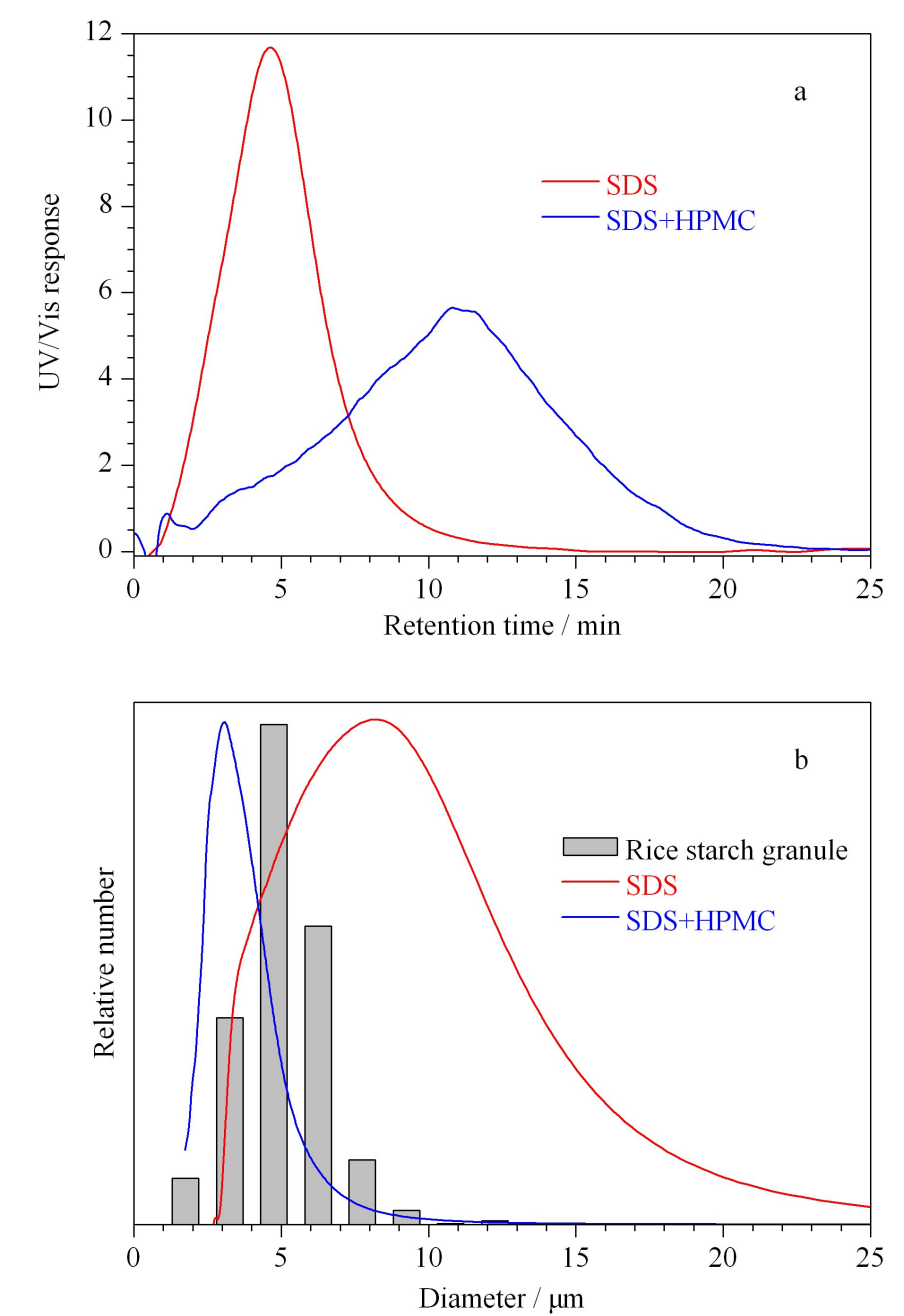
大米淀粉的(a)AF4洗脱谱图及(b)粒径数均分布

### 2.3 AF4对淀粉分子尺寸分布的表征

淀粉颗粒的溶解程度影响淀粉分子尺寸分布和摩尔质量表征的准确性^[19]^。本实验采用碱溶法溶解淀粉颗粒,考察了温度对淀粉颗粒溶解的影响(见图5)。实验结果显示,溶解温度为75 ℃时,红薯淀粉回转半径(*R*_g_)分布和重均摩尔质量(*M*_w_)分布在*t*_r_=7~13 min处出现凸起,这可能是由于红薯淀粉未全溶解,发生了共洗脱现象,粒径大的颗粒与粒径小的颗粒同时洗脱出来^[20,21]^。当溶解温度为78 ℃时,共洗脱现象消失,红薯淀粉溶解较好。由图5a可见,在*t*_r_=5.4 min和*t*_r_=12 min出现两个洗脱峰,前者是直链淀粉洗脱峰,后者为支链淀粉洗脱峰。当溶解温度升高至80 ℃时,AF4-dRI信号的第一个洗脱峰信号增强,第二个洗脱峰信号降低,表明大米淀粉溶解过程中支链淀粉发生降解。综上,选择78 ℃为红薯淀粉的溶解温度。莲子淀粉和大米淀粉的最佳溶解温度为75 ℃。在最佳溶解温度下得到的大米淀粉、红薯淀粉和莲子淀粉的*R*_g_分布范围分别为20~200 nm、20~240 nm、40~200 nm, *M*_w_的分布范围分别为1.80×10^5^~3.13×10^8^ g/mol、1×10^6^~5×10^8^ g/mol、5×10^5^~5×10^8^ g/mol。

**图5:**
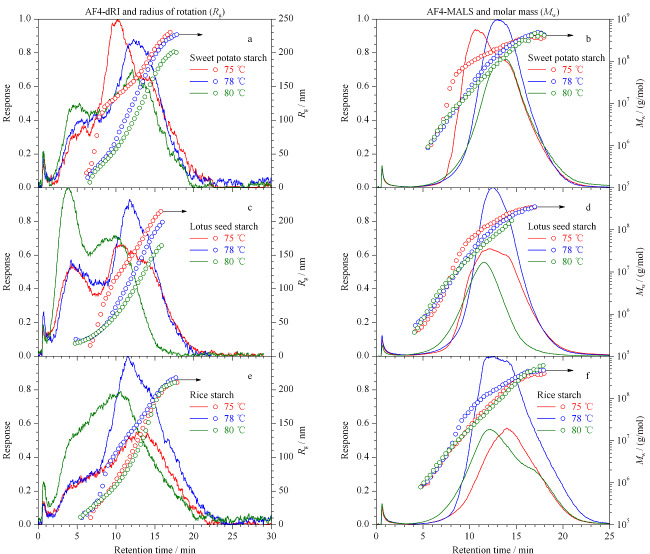
不同淀粉溶解温度下的AF4-MALS-dRI洗脱谱图、*R*_g_和*M*_w_分布图

在图5条件下,对3种淀粉的*R*_g_/*R*_h_和表观密度进行表征(见图6)。*R*_g_/*R*_h_比值在1.0~1.5范围表现为多分支结构^[22]^。实验结果显示,在摩尔质量10^6^~10^8^范围内,大米淀粉的*R*_g_/*R*_h_的比值在1.2~1.4之间,红薯和莲子淀粉的*R*_g_/*R*_h_的比值在0.9~1.1之间。另外,3种淀粉随着摩尔质量的增加,表观密度均呈下降趋势,说明3种淀粉分子随着摩尔质量的增加,结构变得疏松。在相同的摩尔质量下,大米淀粉的表观密度最大,表明其结构更为紧密。

**图6:**
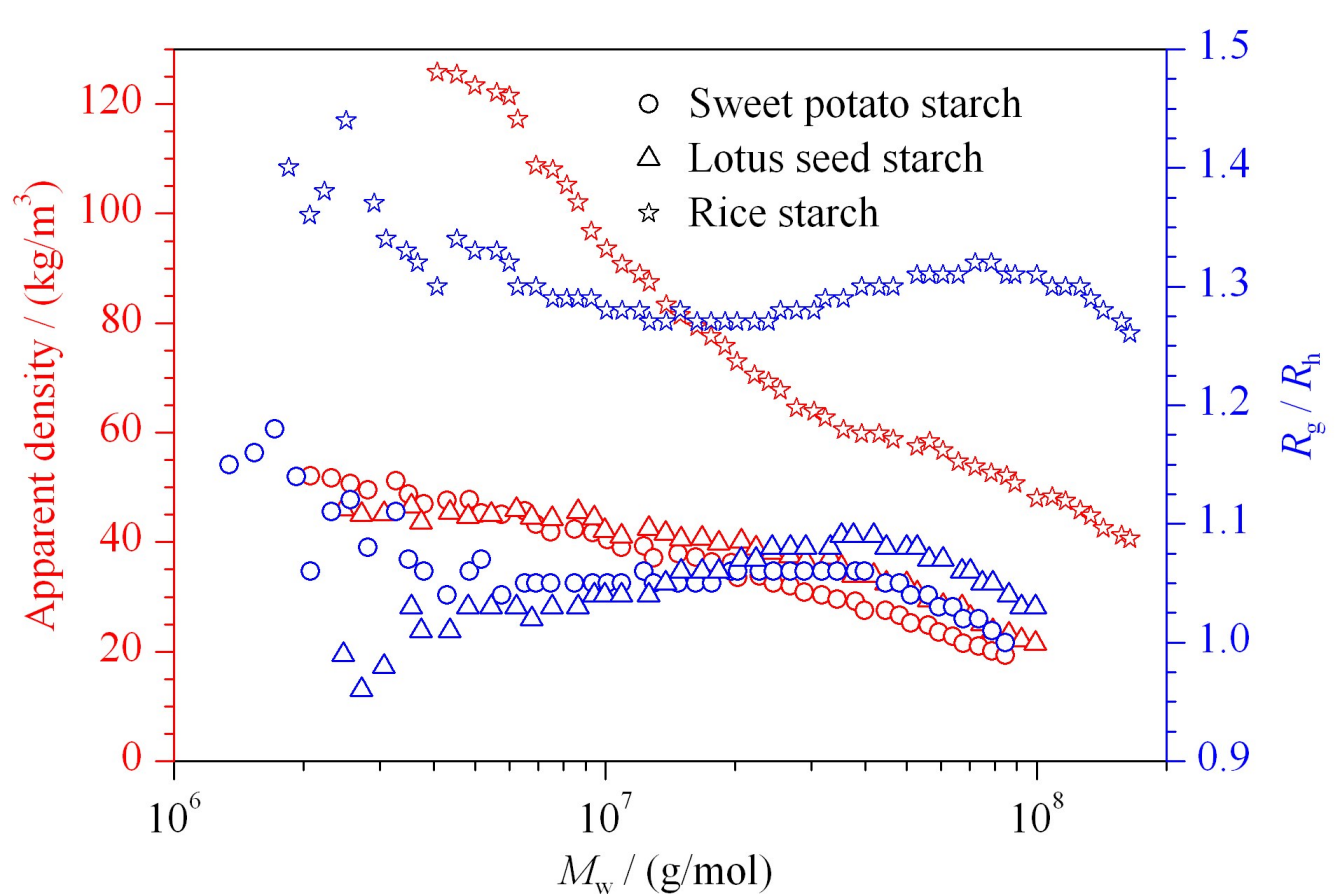
淀粉的表观密度和*R*_g_/*R*_h_分布图

## 3 结论

本研究构建了AF4系统,并对于大米、红薯和莲子3种植物来源不同的淀粉颗粒进行了分离及粒径表征。PS标准样品证明了构建的AF4系统具有良好的分辨率;OM粒径表征结果验证了构建的AF4在微米级淀粉颗粒粒径表征中的准确性。结果显示,载液组成(例如表面活性剂和黏度)影响淀粉颗粒的AF4分离表征结果。商品化的AF4和MALS、dRI联用可表征淀粉分子尺寸及结构。研究结果发现,不同来源的淀粉溶解温度不同,结构有所差异。实验证明了构建的AF4结合商品化的AF4系统,可在纳米至微米范围对淀粉进行分离及粒径表征。
